# Zinc and Lead Metallurgical Slags as a Potential Source of Metal Recovery: A Review

**DOI:** 10.3390/ma16237295

**Published:** 2023-11-23

**Authors:** Katarzyna Nowińska, Zdzisław Adamczyk

**Affiliations:** Faculty of Mining, Safety Engineering and Industrial Automation, Silesian University of Technology, ul. Akademicka 2, 44-100 Gliwice, Poland; zdzislaw.adamczyk@polsl.pl

**Keywords:** metallurgy, zinc, lead, slag, metal recovery

## Abstract

This article presents the mineralogical and chemical characteristics of zinc and lead smelting slags, with particular reference to the slags formed during the simultaneous production of Zn and Pb by the Imperial Smelting Process. These slags, because of the presence of many metals in their composition, mainly in the form of crystalline phases, are a valuable source for their extraction. Slags from Zn-Pb metallurgy are processed on an industrial scale using pyrometallurgical and hydrometallurgical methods, alongside which a number of experiments conducted to recover metals as efficiently as possible, including bioleaching experiments.

## 1. Introduction

Metallurgical slags resulting from zinc and lead production processes are characterised by a diversity of technical parameters, chemical and mineral composition, which in turn depend on the type of feedstock used, i.e., zinc–lead concentrates (type of bed), additives used in the processing (e.g., fluxes), the technological process applied and its course.

Currently, two methods are used to obtain zinc and lead, the pyrometallurgical method and the hydrometallurgical method. This article discusses in detail the slags formed in the pyrometallurgical Imperial Smelting Process (ISP), the target products of which are both Zn and Pb. The chemical and mineral composition of slags from Zn-Pb metallurgy depends on the type of feedstock and on the process conditions.

The process produces two types of slag: shaft furnace slag and refining slag. Shaft furnace slag, which is a waste product of the shaft furnace process, due to its physical and chemical properties, especially its high mechanical strength and low solubility in water, is widely used in road construction, for backfilling in the mining industry and for capping waste dumps with an insulating layer [1,2,3,4,5].

Refining slags, resulting from the lead refining process, contain numerous elements (including toxic metals) and are therefore classified as hazardous waste and deposited in landfills, posing a potential threat to the environment [6,7], in particular to soil and water. On the other hand, refining slags contain numerous strategic and scarce metals and can provide a valuable source of these materials.

The choice of the best slag processing technology is determined by the mineral and chemical composition of the slag.

## 2. Zn-Pb Extraction Technologies

Lead is mainly extracted by pyrometallurgical processing of primary sulphide ore [2,8,9]. Pyrometallurgical methods involve recovering materials (especially metals) at sufficiently high temperatures by transferring them to specific condensed phases (including metallic alloys) or the gas phase with subsequent condensation. Pyrometallurgical methods are chemical processes that take place due to the heat from burned fuel or other exothermic reactions. Obtaining metals from these methods is based on the reduction of metal oxides, most often using carbon (C) or carbon monoxide (CO) [8,9].

Two basic pyrometallurgical processes are used to obtain lead from lead (II) sulphide or mixed concentrates of lead sulphide and zinc sulphide: sintering/smelting (Imperial Smelting Process, ISP) or direct smelting. These processes can also be applied to concentrates containing secondary raw materials.

Zinc is produced by hydrometallurgical and pyrometallurgical methods, which account for 80% and 20% of global production, respectively [7,8,9,10,11]. Hydrometallurgical methods involve leaching a roasted concentrate with an H_2_SO_4_ solution. The zinc sulphate solution obtained by leaching is purified from admixtures, and zinc is then separated from the purified solution in electrolytic tanks. These methods allow high-quality metal production with much lower environmental impact than that of pyrometallurgical methods. However, hydrometallurgical methods cannot be used for lead production because lead salts are only weakly soluble [12,13,14,15].

The most widely used pyrometallurgical method for obtaining zinc is the ISP, which is a highly efficient technique that allows for the processing of complex polymetallic raw materials that cannot be processed by other methods [8,9,10,11]. This approach enables the production of not only zinc but also crude lead. The process is based on the reduction of roasted zinc–lead concentrate with coke [16,17,18].

**Figure 1:**
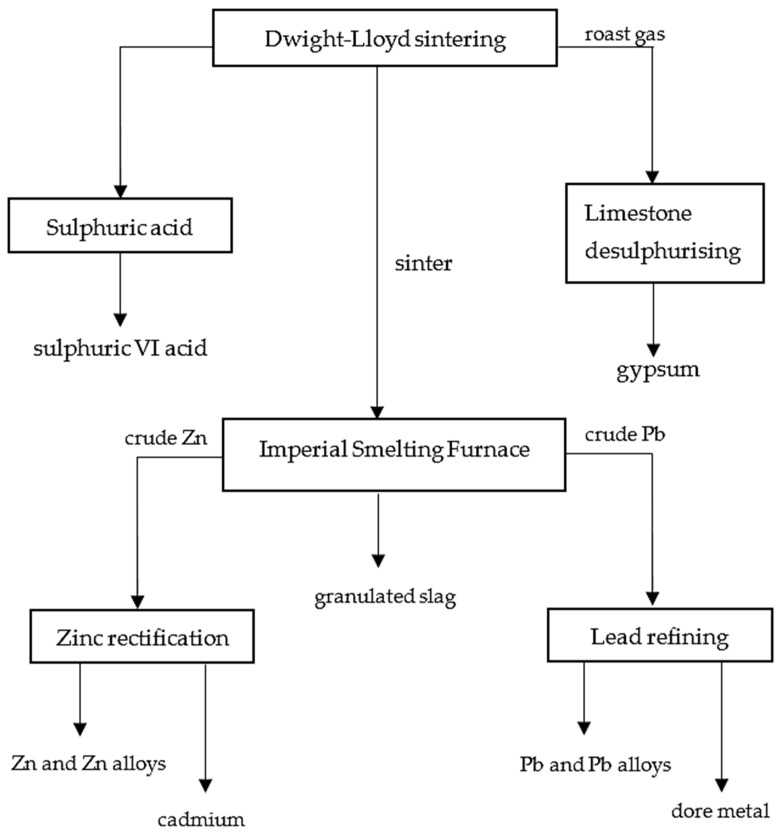
Imperial Smelting Process flowsheet [19].

The process line of a typical smelting plant comprises the following components (Figure 1):

– Sintering unit;
– Shaft furnace unit;
– Lead refining unit;
– Zinc rectification unit.

The feedstock for the ISP process is zinc–lead concentrates and Zn- and Pb-containing oxide waste materials (in-house recyclable waste, i.e., sludge, dust, dross, secondary raw materials—zinc alloy scrap, hard zinc, and foreign crude lead; imported waste, including dust from steel making, zinc dust and dross, zinc sludge, lead oxide, cable scrap (leaden cables), hard zinc waste).

The charge mixture is subjected to an oxidative roasting process at 1200 °C in a Dwight–Lloyd (DL) sintering machine. The products of this process are zinc–lead sinter and post-reaction gas. The gas is transferred to a sulphuric acid production line while the sinter, after crushing and segregation, forms the feedstock for the shaft furnace process [19].

Sinter (and zinc scrap intermittently), heated coke, and lime (as a flux) are automatically fed into the shaft furnace according to a predetermined weight ratio. However, to ensure optimal performance of the reduction process, the ratio of coke to sinter introduced into the furnace, determined by the C/Zn ratio, ranges from 0.8 to 1.2. Upon exposure to hot air blown into the furnace at 1000 °C, the coke is burned to produce CO, which serves as the reducing agent in the process [16,17,18].

The reduction process occurs in three distinct zones in the furnace shaft:

– upper zone—reduction of lead oxide:

PbO_(s)_ + CO_(g)_ = Pb_(l)_ + CO_2_ (1)

– middle or equilibrium zone—Boudouard reaction:

CO_2(g)_ + C_(s)_ = 2CO_(g)_ (2)

– lower zone—reduction of zinc oxide:

ZnO_(s)_ + C_(s)_ = Zn_(g)_ + CO_(g)_ (3)

The essential sinter melting processes take place in the lower part of the shaft furnace in the melting and reduction zone, where zinc and lead oxides are reduced, and the slag is liquefied. Lead, liquid slag with a melting point of around 1200 °C and process gases containing CO_2_, CO, N_2_, and Zn vapours are formed in the melting zone. The zinc vapours discharged from the furnace, along with the post-reduction gases, are fed into a condenser where they are condensed in splashed liquid lead circulating in a closed loop. The lead with dissolved zinc is pumped from the condenser to a separation system where the zinc condenses. When the temperature is lowered to 450 °C, the zinc is separated from the lead. From the separation system, the zinc is then transferred to a zinc tank and subsequently to the rectification column feed furnaces [16,17,18].

Slag and lead are discharged at intervals from the bottom of the shaft furnace into a settling tank, where the lead is separated from the slag. After segregation, the slag is directed into a granulation trough, and the residual lead is poured into a ladle and cast into blocks of bullion lead, which are then sent to a lead refinery [19].

**Figure 2:**
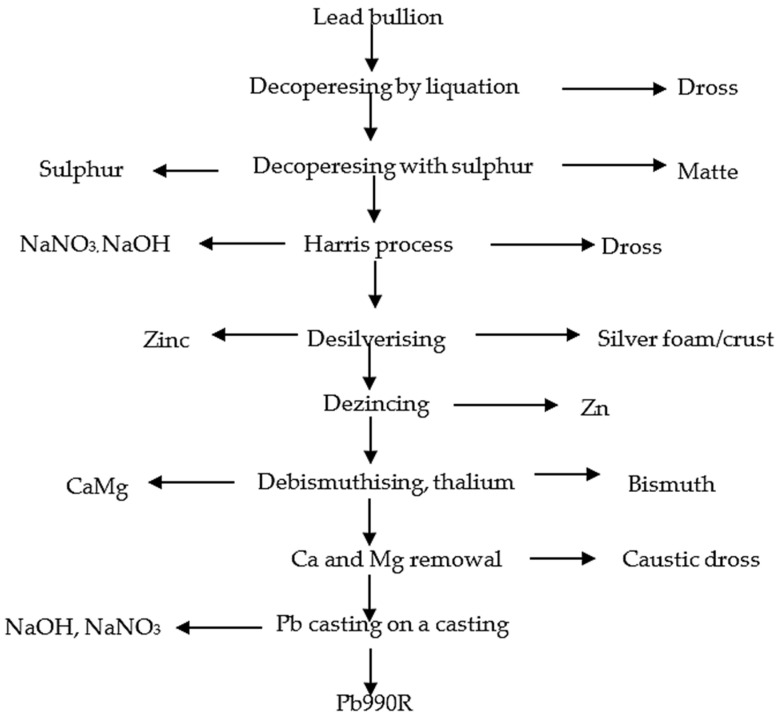
Thermal lead refining flowsheet [19].

Lead refining is based on a thermal process carried out in 13 refining boilers and a short rotary kiln. The refining step aims to separate the precious metals contaminating the lead bullion as efficiently as possible and purify it of admixtures. To separate them from the lead, minor constituent properties are used such as decreasing solubility with decreasing temperature (Cu, intermetallic phases), ability to form lead-insoluble intermetallic phases (Ag, Bi), and higher chemical affinity to oxygen (Sn, As, Sb, Zn, Ca, Mg). Precious metals (Ag, Au) are removed using the Parkes process, which exploits the ability of alloyed zinc to mix with lead, leading to the formation of a so-called silver froth or silver crust, which is then processed in a liquation muffle and distillation furnace to produce a Pb-Ag alloy containing approximately 20–40% silver. Refined lead is cast using a casting machine into ingots which form the final product (Figure 2).

## 3. Slags Formed in Zn-Pb Metallurgy

### Chemical and Mineral Composition

The main chemical components of metallurgical slags formed directly during Zn and Pb processing include FeO_total_, SiO_2_, Al_2_O_3_, CaO, MgO, ZnO, and PbO [3,19,20,21,22,23,24] (Table 1). These slags are characterised by significant variability in the content of their main constituents, with ranges including FeO_total_ 0.88–59.6 wt%, SiO_2_ 2.04–57.1 wt%, CaO 0.18–32.23 wt%, MgO 0.61–15.9 wt%, ZnO 0.03–47.3 wt%, and PbO 0.002–6.4 wt% (Table 1).

Refining slags exhibit different chemical compositions from slags from the shaft process of Zn-Pb metallurgy. Refining slags are dominated by ZnO (average content 12.2 wt%), PbO (average content 17.5 wt%), CuO (average content 11.5 wt%), and SO_3_ (average content 13.6 wt%), whereas the average content of these components is several times lower in slags from the shaft process (Table 1). Like slags from the shaft process, their composition also varies considerably (Table 1).

In addition to the main constituents represented in oxide form, Zn-Pb metallurgical slags contain numerous minor constituents, including As, Ba, Cd, Co, Cr, Cu, Ni, Sb, Sn, and V, the concentrations of which also vary within a wide range (Table 1).

The variation in the chemical composition of slags taken from different layers results from several factors, some of which include the variability in the feedstock for the lead refining process, the conditions involved in the industrial process, and weathering processes occurring in the landfill body (in the case of landfilled slags).

Due to the great variety of forms of mineral constituents, which usually occur as multiphase conglomerates with numerous admixtures, it is extremely difficult to determine the phase composition of metallurgical slags.

**Figure 3:**
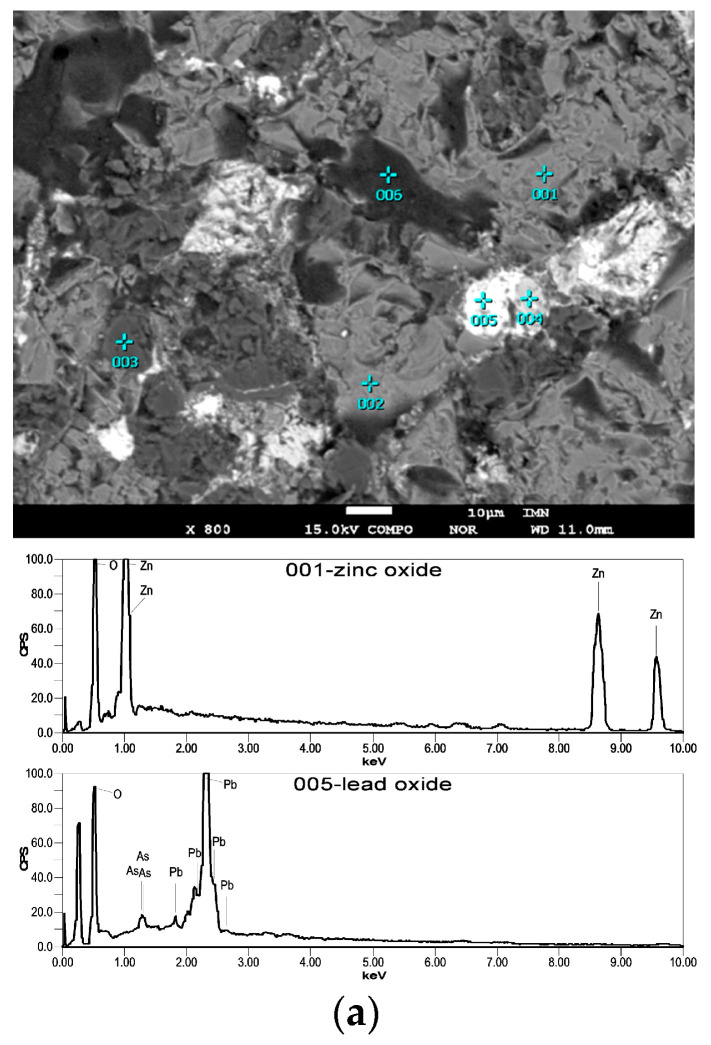

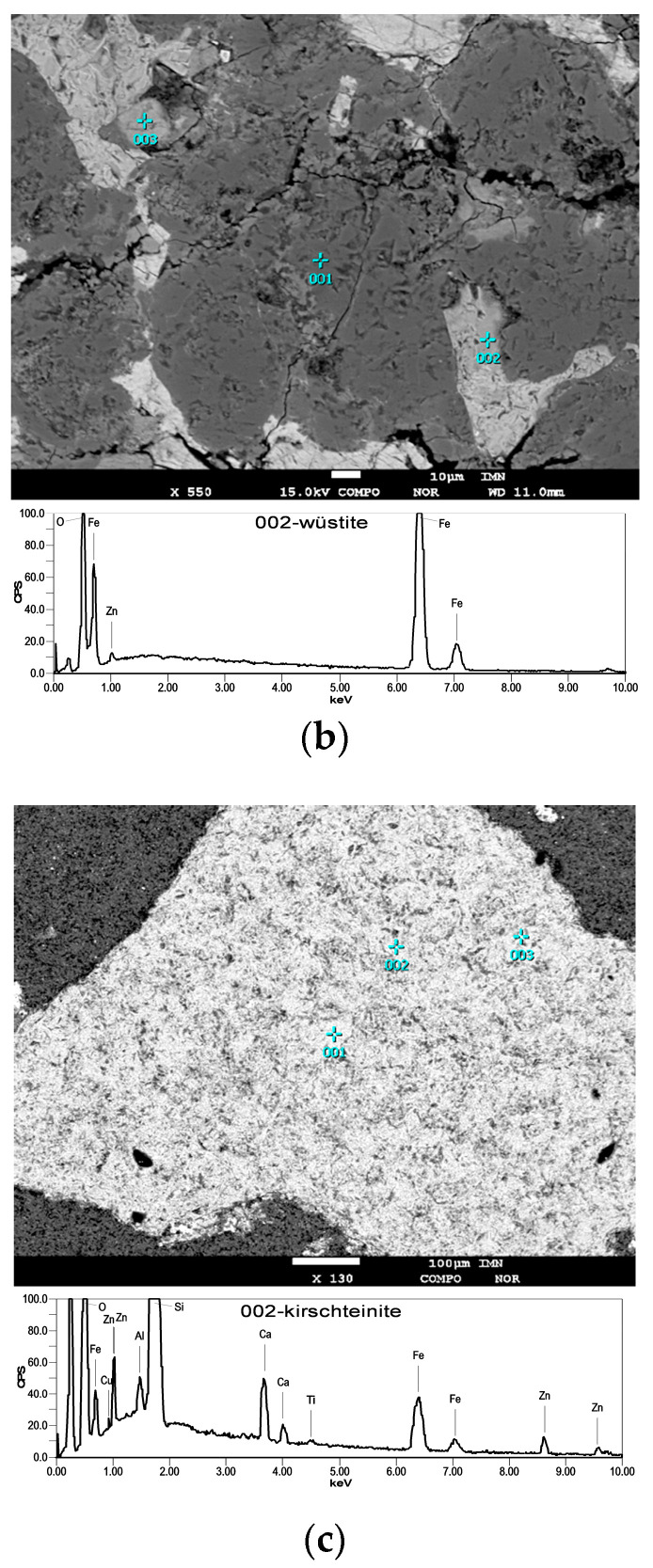
Examples of microareas of Pb refining slag samples [19]: (**a**) example of an image of the investigated microareas: 001- and 002-wüstite and zinc oxide in glass, 003-willemite, 004-alamosite, 005-lead oxide, 006-quartz [19]; (**b**) example of an image of the investigated microareas: 001-Na_2_Zn (Si_2_O_6_) with impurities of wüstite, 002-wüstite, 003-alloy type Pb_27_Fe_24_Zn_10_Cu_10_O_16_C_7_Rs_7_ [19]; (**c**) example of an image of the investigated microareas: 001- and 002-kirschteinite with Na_2_Zn(Si_2_O_6_) and carnegieite with admixtures of sphalerite, 003-Ag met [19].

The phase composition of Zn-Pb metallurgical slags varies greatly, and the most common mineral constituents are presented in Table 2 (Figure 3). The phase composition of slags also includes metallic precipitates (Zn, Pb, Cu, Fe) and multicomponent metal alloys (Pb, Zn, Cu, Fe, As, Sb) doped with numerous elements: Sn, Bi, Tl, Na, S, Fe, Cd, Sn, Ti, Ca.

In Zn-Pb metallurgical slags, in addition to the crystalline components, there is also amorphous glass, the content of which depends on the cooling rate of the slags. The glass content of rapidly cooled slags is significantly higher than that of slags cooled slowly, e.g., in an air atmosphere. Quantifying glass is highly challenging due to its amorphous nature and varying SiO_2_ content, resulting in an ambiguous interpretation of the reflections related to the corresponding d_hkl_ values in X-ray structural studies [27,44]. The glass also contains elements such as Fe, Al, Ca, Pb, Zn, Cu, and As, which occur in the form of nanometric oxide inclusions and intermetallic compounds [3,19,27,44].

In classifying the phase constituents of the Zn-Pb pyrometallurgical slags, the following constituents were distinguished: those originating from the technological process (silicates: carnegieite, chalcocite, olivine, and kirschsteinite; sulphate: anglesite; oxides: wüstite, ZnO, PbO; hydroxide: alamosite), those crystallising under hypergenic conditions in landfill (sulphates and hydrated sulphates: ktenasite, namuvite, and posnjakite; oxides and hydroxides: tochilinite, goethite, and gerhardtite; carbonates: cerussite, calcite), and those with the nature of feedstock minerals (mainly the sulphides ZnS and PbS) [19].

## 4. Processes for the Recovery of Metals from Zn-Pb Metallurgical Slags

Metallurgical slags that contain metals at concentrations of several per cent can serve as a source of metals. The mineral and chemical composition of metallurgical slags determines their processing method. Due to the variety of forms of metal occurrence in slags, it is extremely difficult to identify the optimal technological process for their processing. Metals from slags (so-called secondary metals) generated in the pyrometallurgical process to obtain Zn-Pb are typically recovered using pyrometallurgical and hydrometallurgical methods.

### 4.1. Pyrometallurgical Methods

Pyrometallurgical processes, sometimes referred to as thermal metallurgy, are based on the processing of slags at high temperatures in various furnace types, including shaft, rotary, electric, and muffle furnaces [45,46,47,48].

#### 4.1.1. Fuming Process

One widely used method of pyrometallurgical processing of metallurgical waste is fuming, which involves recovering zinc and lead from liquid slag blown with air and coal dust or natural gas to provide a reducing atmosphere. The metals contained in the slag are reduced and evaporated and then re-oxidised (Table 3) [47,48,49,50,51,52].

This process follows these summary chemical equations [47,48]:

(ZnO)_slag_ + CO = Zn_(g)_ + CO_2_ (4)

(Cd)_slag_ + CO = Cd_(g)_ + CO_2_ (5)

(PbO)_slag_ + CO = Pb_(g)_ + CO_2_ (6)

(Fe_2_O_3_)_slag_ + CO = 2(FeO)_slag_ + CO_2_ (7)

C + CO_2_ = 2CO (8)

These reactions take place at a temperature of around 1500 °C. Fuming is usually carried out in cyclone furnaces or converters.

Spent gases containing a mixture of metal oxides are cooled, and the dust is retained in bag filters. The resulting dust, with a content of 60–75% Zn and 15–25% Pb, is then processed either pyrometallurgically (ISA furnaces) or hydrometallurgically (electrolysis). The waste material of the process is slag with a Zn content of 1.5–2.5% and a Pb content of ~0.2%, which is then granulated or cast into slabs used for the manufacture of construction aggregates [47,48] (Table 4).

#### 4.1.2. Isasmelt Process

The second most common pyrometallurgical method involves remelting lead metallurgical slags in Isasmelt and Kaldo furnaces. These furnaces are used to process both primary and secondary raw materials, such as zinc electrolysis slurries, zinc-bearing slags, EAF dust, and various metallurgical waste types (Table 5). Fuel and process gases are fed into the furnace via a lance directly below the surface of the liquid slag, which provides highly turbulent conditions favourable for the mass and heat transfer processes.

**Figure 4:**
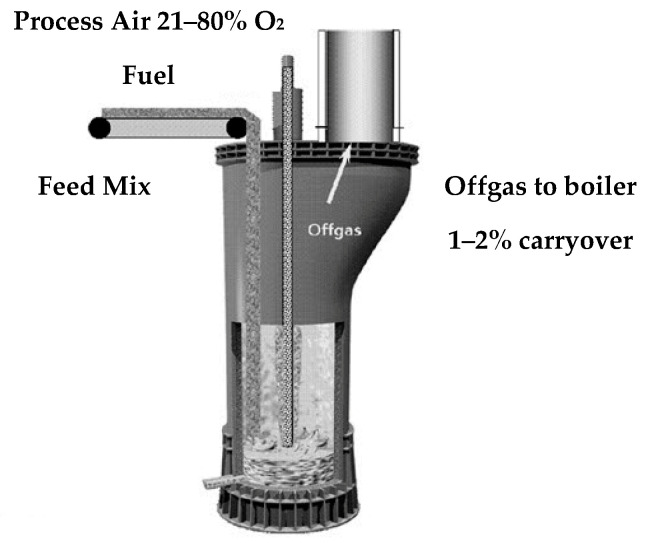
Scheme of Isasmelt technology [54].

The design of the Isasmelt furnace and the use of a steel lance allow the melting, oxidation and reduction processes to be performed. The remelting process is carried out at a temperature of 1150–1250 °C [47,48,53] (Figure 4).

To increase the efficiency, this operation is split between two furnaces. In the first furnace, a continuous melting process is performed, with oxidation conducted by air injected through a lance. The lead obtained in the process is tapped out of the furnace via a siphon, while the lead-bearing slag is transferred intermittently to the second furnace, in which the lead is reduced with coal, and, after the lead is drained, slag fuming is performed. The end product of the process is dust containing 46–58% Zn, 18–30% Pb, and 0.1–2.7% Cd in addition to waste slag [54,55,56,57,58,59].

#### 4.1.3. Kaldo Process

Kaldo furnaces do not perform charge sintering as a separate step in the process. Instead, the secondary materials together with lead sulphide concentrate (dried to a moisture content of <1% and a particle size of <2 mm) are fed directly into the furnace and then melted at 1400 °C and oxidised. The Kaldo furnace is a tilting rotary vertical converter equipped with a system of three concentric lances: the inner lance is used to deliver the charge, the middle lance delivers the fuel, and the outer lance delivers air and oxygen [47,48,53].

#### 4.1.4. Electric Furnace Process

Another metal recovery approach, particularly to copper recovery from the slags of nonferrous metallurgy, is the traditional method of reducing liquid slags in an electric furnace using reducing and sulphurising agents such as coal, carbide, pyrite, and pyrrhotite in addition to reducing gases. The recovery rates of copper and other metals using this method are below 90%, and the disadvantages of this method include its significant energy consumption, long process duration, and the need for reducing agents [60].

#### 4.1.5. Thermal Electrolysis

Another method of copper recovery from Zn-Pb metallurgical slags involves subjecting the molten slag to thermal electrolysis, which is carried out collectively (i.e., in the same furnace) or selectively in several units, where metallic alloys of different compositions are obtained by applying different current–voltage conditions [47,48,61]. Copper recovery in this approach is carried out by fitting graphite electrodes that supply a direct current. The flow of current through the liquid slag results in electrolytic separation of the metals dissolved within it, in addition to the separation of metallic precipitates by electrocapillary movement in the electric field. The physical and chemical properties of the slag are adjusted, depending on its composition, by introducing suitable additives, such as calcium fluoride, sodium chloride, or sodium carbonate [47,48,61]. This process yields Cu (>90%) and other accompanying metals, e.g., Pb, Zn, and Ni, in the form of an alloy that can be further processed [47,48,61].

#### 4.1.6. Black Sea Copper Works Process

An innovative process for recovering copper from slag has been developed at the Black Sea Copper Works (Turkey), in which the slag is cooled in air for 24 h and then crushed and ground until 80% is a fraction finer than 0.1 mm; this fraction is subsequently floated to produce a flotation concentrate for remelting.

**Figure 5:**
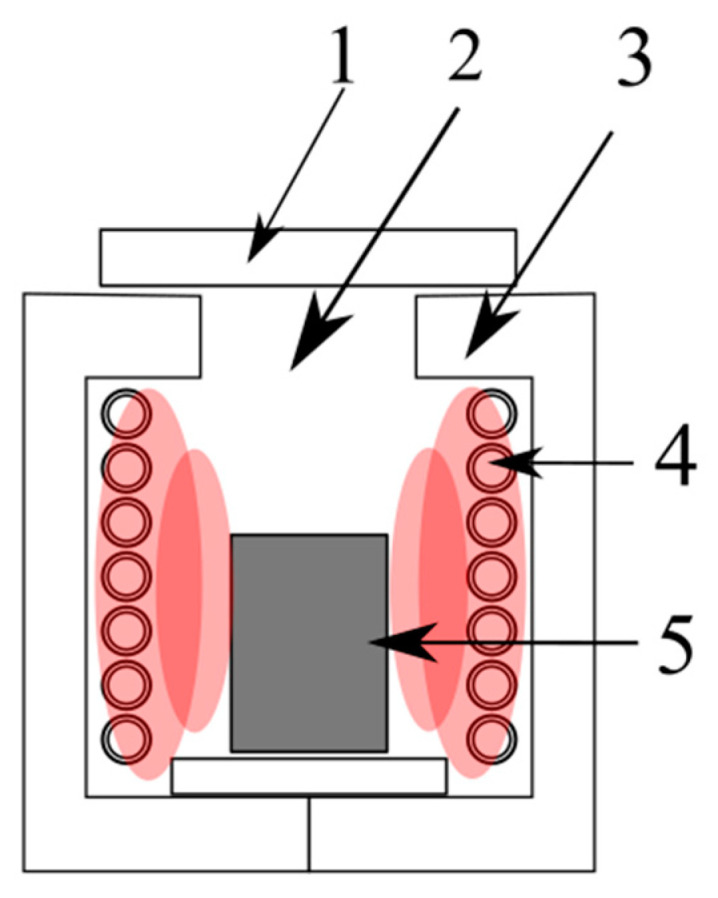
Scheme of the soaking furnace: (1) furnace cover, (2) heating chamber, (3) furnace casing, (4) heating elements made of Kanthal wire, (5) crucible [19].

Various other methods have been developed for metal recovery from slags; however, most of them have only been tested on a laboratory scale. An example of an attempt to recover metals from Zn-Pb metallurgical slags at a laboratory scale involves processing a refining slag in a resistance pit furnace at 1250 °C (Figure 5) with a pre-oxidation roasting step conducted by blowing 50 dm^3^/h of air through the slag at 800 °C to allow the following reactions to proceed:

ZnS + 1.5O_2_ → ZnO + SO_2_ (9)

PbS + 1.5O_2_ → PbO + SO_2_ (10)

It is mainly these reactions that take place in the workspace of the furnace:

2ZnO + C → Zn_g_ + CO_2_ (11)

ZnO + CO → Zn_g_ + CO_2_ (12)

2PbO + C → Pb + CO_2_ (13)

PbO + CO → Pb + CO_2_ (14)

CaCO_3_ → CaO + CO_2_ (15)

2Zn_g_ + O_2_ → 2ZnO (16)

**Figure 6:**
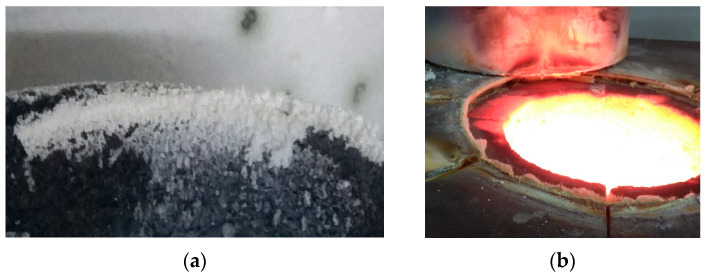
Zinc oxide depositing (**a**) on the filter and (**b**) in the furnace chamber [19].

In this process, the furnace charge consists of refining slags, coal (reducing agent), and limestone CaCO_3_ (flux). During the processing of the slags, sample weight loss was observed, which primarily occurred due to zinc evaporation caused by the reduction process. This zinc then is oxidised and accumulates on the filter in the form of ZnO (Figure 6a) [19]. In addition to the zinc oxidation reaction, the observed change in the sample weight in this method may result from various other factors, including the reduction of lead oxide, the combustion of coal and sulphur, or the addition of limestone or iron. The lead yield in this process is higher than 75%. It is difficult to determine the efficiency of zinc recovery from slags because during their processing, Zn is oxidised, and the resulting ZnO accumulates not only on the filter but also in the furnace chamber (Figure 6b) [19]. This ZnO would require further leaching with a sulphuric (VI) acid solution to obtain Zn.

### 4.2. Hydrometallurgical Methods

#### 4.2.1. Chemical Leaching

Hydrometallurgical methods of metal recovery from waste include leaching, solution purification, metal separation, and isolation of pure metals at temperatures below 100 °C.

The leaching process, which forms the basis of hydrometallurgical processes, can be performed using acid solutions (such as sulphuric or hydrochloric acid), ammonia solutions, or alkaline hydroxide solutions. The choice of the leaching medium depends on the chemical form in which the metal is present in the waste [47,48,62,63,64,65,66,67]. Most studies to date have investigated the leaching of Zn-Pb metallurgical waste using sulphuric (VI) acid solutions, with the choice of leaching conditions dependent, among other factors, on the mineral and chemical composition of the metallurgical waste, as the solubility of its constituent phases in sulphuric (VI) acid solutions varies significantly [47,48,62,63,64,65,66,67]. Depending on the form of zinc present, the zinc leaching process occurs according to the equations exemplified below [47,48]:

ZnO + H_2_SO_4_ = ZnSO_4_ + H_2_O (17)

ZnFe_2_O_4_ + 4H_2_SO_4_ = ZnSO_4_ + Fe_2_(SO_4_)_3_ + 4H_2_O (18)

Fe_3_O_4_ + 4H_2_SO_4_ = FeSO_4_ + Fe_2_(SO_4_)_3_ + 4H_2_O (19)

Ca[Zn(OH)_3_]_2_ · H_2_O + 3H_2_SO_4_ = CaSO_4_ + 2ZnSO_4_ + 8H_2_O (20)

ZnSiO_3_ + H_2_SO_4_ = ZnSO_4_ + SiO_2_ + H_2_O (21)

The rates of the above reactions are determined by several factors, the most important of which are the mineral composition, material grain size, and sulphuric (VI) acid concentration. Zinc silicate (ZnSiO_3_) solubilises at a much lower rate than the oxide form (ZnO), while the efficiency of the leaching process following Reactions (17) and (19) increases significantly with increasing temperature. Other slag components, i.e., Cu, Ni, Cd, Pb, etc., react similarly to sulphuric (VI) acid.

Minor components present in the zinc-bearing material can be classified into two groups [47,48]:

– Those passing into solution during the leaching process, including Cu, Cd, Fe, Mg, and Ni;
– Those forming insoluble salts, including PbSO_4_, CaSO_4_∙2H_2_O, BaSO_4_.

The next step in the zinc recovery process is the purification of the zinc sulphate (VI) solution, which is then electrolysed. In practice, chemical purification of the electrolyte may be carried out continuously or in several stages. The average composition of the crude ZnSO_4_ solution is 135 mg/dm^3^ Zn, 470 mg/dm^3^ Cu, 168 mg/dm^3^ Cd, 18 mg/dm^3^ Fe, 0.9 mg/dm^3^ As, 0.11 mg/dm^3^ Sb, and 12.7 mg/dm^3^ Co [47].

Purification of the zinc sulphate solution is conducted using various methods, which are selected according to the type of contaminant to be removed. Some impurities (As, Sb, Fe) are removed during neutral leaching, as the salts of these elements hydrolyse under these conditions and are adsorbed onto the Fe(OH)_3_ surface. Hydrolytic purification involves selecting suitable process conditions (solution pH, temperature, etc.) to ensure that the usually sparingly soluble hydroxides of metals that contaminate the solution are formed.

Depending on the conditions of the hydrolytic electrolyte purification process, iron precipitates from the solution in the form of goethite (FeOOH), hematite (Fe_2_O_3_), or jarosite (MFe_3_(SO_4_)_2_(OH)_6_, where M = NH^4+^, K^+^, Na^+^, or 0.5Pb^2+^). The preferred precipitate type in hydrometallurgical processing is jarosite. The obtained jarosite is characterised by highly favourable filtration properties, which significantly reduce operating costs for the process. In addition, during jarosite precipitation, it is also possible to remove other impurities from the electrolyte, e.g., arsenic [47,48]. To remove copper, cadmium, nickel, and cobalt from the zinc sulphate (VI) solution, the cementation method (internal electrolysis) is applied, which involves precipitating the more noble metal from the electrolyte with zinc dust.

Ammonia solutions are another group of agents commonly used for metal leaching in hydrometallurgical processes. A Spanish company, CENIM, and a Portuguese company, LENTI, developed a CENIM–LENTI technology to process sulphide concentrates and zinc-bearing waste using ammonium salts. The main waste-leaching reagent in this process is ammonium chloride. Zinc in the leach residue is present as ZnO-Fe_2_O_3_, which is then extracted with a solution of di-2-ethylhexylphosphoric acid (D2EHPA) and isolated using electrolysis [47,48,53,68,69].

PbCl_2 (s)_ + 2Cl^−^ _(aq)_ → PbCl_4_^2−^ _(aq)_ (22)

A study using acetic acid as the leaching agent for Pb from slags was performed by Forte et al. [70]. In their study, the leaching process involved dissolving metallic Pb in concentrated acetic acid and then precipitating PbSO_4_ by adding H_2_SO_4_ to the solution. However, the major disadvantage of this method is that only lead present in metallic form is recovered.

**Figure 7:**
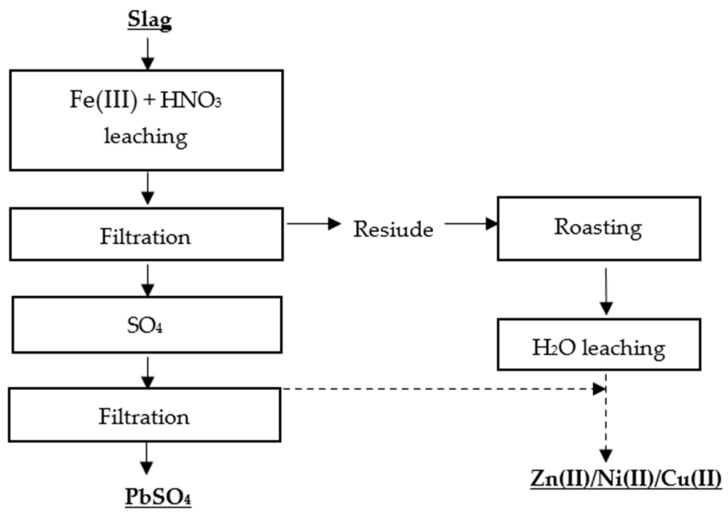
Flowsheet of two-stage leaching of Pb, Zn, Ni, Cu [71].

**Figure 8:**
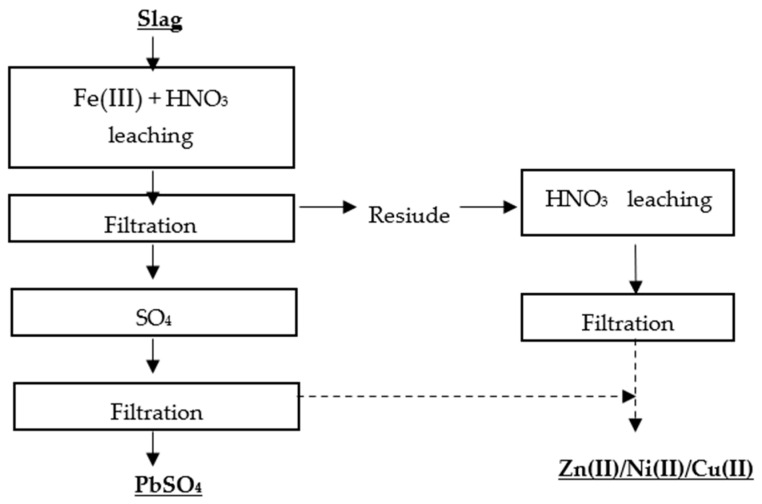
Flowsheet of single-stage leaching of Pb, Zn, Ni, Cu without roasting [71].

Kim et al. [71] proposed two methods for the selective recovery of lead, copper, nickel, and zinc from lead slag. The first metal recovery method was based on a two-stage leaching process, in which the first stage involved leaching the metals with Fe(III) + HNO_3_, and the second stage involved roasting the residue and leaching it with water (Figure 7 and Figure 8).

Attempts have also been made to process hydrometallurgical waste using sodium hydroxide. The main problem with this method is the presence of sparingly soluble zinc compounds in the waste, e.g., ferrites.

Leaching of zinc from metallurgical waste has been conducted using NaOH solution under various conditions. This process was most commonly performed at temperatures of 25 and 90 °C, using NaOH solutions of 2–6 mol/dm^3^ concentration for 4 h (traditional method). Pressure leaching in an autoclave using a 6 mol/dm^3^ NaOH solution at 120–200 °C for 4 h was also investigated, as was the leaching process after pretreating the waste in a microwave oven (1 kW, 2.45 GHz). The best results were obtained with the traditional method, in which the zinc recovery reached 74%. In addition to iron oxides, the residual solid phase after leaching contained insoluble zinc ferrites [72,73,74].

#### 4.2.2. Bioleaching

Another group of methods used to recover metals from metallurgical slags involves bioleaching, a process in which microorganisms are used to convert solid, insoluble metals and their compounds to water-soluble forms [75,76]. Most microorganisms capable of biohydrometallurgical processes belong to the group of chemolithotrophs, which use carbon dioxide as their source of cellular carbon. For their energy source, they can use reduced sulphur and iron compounds or oxidation reactions of elemental sulphur, sulphides, or thiosulphates.

The microorganisms involved in leaching include not only bacteria (genera: *Acidithiobacillus*, *Thiobacillus*) but also fungi (including the genera *Penicillium*, *Aspergillus*, *Fusarium*, *Alternaria*, and *Candida*). In practice, biohydrometallurgical processes are carried out using mixtures of microbial populations that occur naturally in iron- and sulphur-rich environments; bacterial monocultures are not used. The bioleaching process follows two main mechanisms: indirect and direct [77,78,79,80].

The indirect mechanism involves chemical and bacterial oxidation, with microbial oxidation of Fe^2+^ ions derived from minerals used to form Fe^3+^ ions, which then participate in the leaching process. Microorganisms are the source of the leaching agent, which chemically oxidises the sulphide minerals. In this model, no physical contact occurs between the bacterial cell and the mineral surface [77,78,79,80].

MeS + Fe_2_(SO_4_)_3_MeSO_4_ + FeSO_4_ + S (23)

S + 3O_2_ + 2H_2_O → 2H_2_SO_4_ (24)

In the direct mechanism, the electrons obtained in the bacterial oxidation process are sourced directly from the reduced minerals. In this model, there is physical contact between the bacterial cell and the mineral surface. These reactions are most often associated with the oxidation of pyrite.

4FeS_2_ + 15O_2_ + 2H_2_O → 2Fe_2_(SO_4_)_3_ + 2H_2_SO_4_ (25)

MeS + 2O_2_ → MeSO_4_ (26)

Microorganism selection is a key factor when performing bioleaching of metallurgical slags. The indigenous microorganisms present at the site where the slags are deposited have the highest contaminant removal efficiency by adapting to conditions with higher metal content. For this reason, *Acidithiobacillus* bacteria are the most commonly used in studies of bioleaching of components from metallurgical slags because environmental bacteria from these genera have been widely reported in slag deposition sites [77,78,79,80].

Laboratory-scale bioleaching experiments include batch leaching, semi-open flow-through leaching, and continuously stirred tank reactors [79,80,81,82,83,84]. On a laboratory scale, a method has been developed to process slags from zinc and lead production from former Yugoslavian plants by gravity enrichment of the slags, resulting in a concentrate with a 94.5 wt% Pb content. Waste from the enrichment process containing 38.7 wt% Fe, 32.3 wt% SiO_2_, 5.8 wt% Zn, and 3.0 wt% Pb is subjected to bacterial leaching using autotrophic thionic bacteria to recover zinc [47,48,61].

Bioleaching can be used not only to extract valuable metals but also to remove toxic elements by disrupting the amorphous structure of slags. The efficiency of bioleaching depends on various factors, including the pH, leaching time and temperature, and slag structure [82,83,84]. However, despite its high efficiency, bioleaching to date has only been performed on a laboratory scale. Due to the long processing times, low yields, and problems with separating metals from the solution associated with this approach, it has not yet been applied at an industrial scale.

## 5. Discussion and Conclusions

This article presents a literature review on metallurgical slags from the zinc and lead production process as a source material for metal extraction. The core conclusions of the article are as follows.

– Metallurgical slags from zinc and lead production contain significant amounts of metals and semi-metals, dominantly Si, Al, Ca, Ma, Zn, and Pb. Among the slags of Zn-Pb metallurgy, refining slags are distinctive due to their much higher contents of Zn, Pb, and Cu and significantly lower concentrations of Si, Al, Ca, and Mg.
– The mineral composition of Zn-Pb metallurgical slags is dominated by multiphase crystalline conglomerates formed by high-temperature processes. These take the form of intergrowths of fine, intercalated clusters of individual phases, among which one is always dominant. Typical mineral constituents in these slags include Zn and Fe oxides, Fe hydroxides, Zn, Pb, Fe and Cu sulphides, Pb sulphates, and hydrated Zn, Ca, Cu sulphates, Zn silicates, olivine group silicates, melilites (Ca,Na)_2_(Al,Mg)[(Si,Al)_2_O_7_], Pb and Zn carbonates, spinels, and multicomponent metal alloys of Pb, Zn, Cu, Fe, As, and Sb. In addition, tochilinite [Fe_0.9_]_6_S_6_[Mg_0.71_Fe_0.29_(OH)_2_]_5_ is present in refining slags [85,86], whereas this component has not been identified in shaft slags from Zn-Pb metallurgy.
– The choice of slag processing technology is determined primarily by the mineral composition of the slag, which in turn is determined by various factors, including the diversity of the feedstock, the variability in the technological parameters, and the rate of slag cooling (which determines the ratio of the amorphous to the crystalline phase). Slag processing methods commonly applied on an industrial scale include pyrometallurgical techniques, such as fuming, slag remelting in Isasmelt or Kaldo furnaces, and thermal electrolysis, and hydrometallurgical leaching, which can be performed using acid solutions (such as sulphuric or hydrochloric acid), ammonia solutions, or alkaline hydroxide solutions. The choice of the leaching medium depends on the chemical form in which the target metal is present in the waste. Various other methods have also been developed to recover metals from slag; however, most of these have only been tested on a laboratory scale. For example, the bioleaching method, despite its high efficiency, has not been applied on an industrial scale due to its long process duration, low yields, and problems with separating metals from the solution.
– The pyrometallurgical and hydrometallurgical methods described in detail in this article are used on an industrial scale due to their high efficiency (amounting to >80% average metal recovery) and effective waste neutralisation by obtaining high-purity metallic products while minimising the formation of toxic secondary waste. Other Zn-Pb slag processing methods described in the literature, i.e., the IBDR-ZIPP pyrometallurgical process or hydrometallurgical processes (e.g., Española del Zinc, Placid), represent technological concepts verified on a pilot scale.
– The presented technologies in this article represent a rational approach to using secondary materials from Zn-Pb metallurgy and are consistent with current circular economy trends. These methods also consider complementary activities aimed at the following:
  - Improving the efficiency of using Zn-Pb slags as a secondary raw material;
  - Comprehensive processing of Zn-Pb metallurgical waste;
  - Reducing the amount of waste generated at individual stages of the technological process.
– The processing of metallurgical slag is an extremely important topic as, in addition to the economic benefits of the process, it significantly neutralises the potential of slag to act as a source of environmental pollution.

## Figures and Tables

**Table 1:** Summary of major chemistry (in wt%) and minor chemistry (in mg kg^−1^) of Zn-Pb metallurgical slags and Pb refining slags [3,19].

Component	Zn-Pb Slags			Pb Refining Slags		
	Min	Max	Average *	Min	Max	Average **
Al_2_O_3_	0.90	21.9	8.3	0.57	7.8	2.8
CaO	0.18	32.2	17.3	0.14	5.65	2.8
FeO_Total_	0.88	59.6	16.7	8.3	31.1	20.0
K_2_O	0.02	3.91	0.60	0.04	0.27	0.15
CuO	-	-	-	0.98	20.93	11.5
MgO	0.61	15.9	5.4	0.10	0.81	1.1
PbO	0.0002	6.4	0.93	0.72	44.6	17.8
ZnO	0.03	47.2	4.93	6.6	18.07	12.2
MnO	0.01	3.0	0.5	0.02	0.55	0.21
SO_3_	0.05	14.9	2.99	5.83	23.1	13.6
SiO_2_	2.04	57,1	28.6	2.45	35.5	11.1
TiO_2_	0.07	1.14	0.4	0.04	0.34	0.16
Element	Min	Max	Average *	Min	Max	Average **
As	1	10,710	1181	147	15,558	8843
Ba	76	17,914	1126	336	778	508
Cd	0.38	575	31.2	85	19,757	5595
Co	8.5	242	35.7	0.01	452	232
Cr	4	700	155	132	708	435
Ni	13	240	59.7	76	447	311
Cu	16	7400	802	-	-	-
P	43.57	26,400	5138	-	-	-
Sb	0.16	245	42.6	81	9869	472
Sn	0.1	500	23.4	0.01	617	323
V	6	9980	2294	-	-	-

* Arithmetic average values according to Piatak et al., 2021 [3]; ** arithmetic average values of 64 samples according to Nowińska, 2022 [19].

**Table 2:** Main phase components of Zn-Pb slags [3,19,25,26,27,28,29,30,31,32,33,34,35,36,37,38,39,40,41,42,43].

Group	Phase	Chemical Formula
Oxides	Zincite	ZnO
	Wüstite	FeO
	Hematite	Fe_2_O_3_
Hydroxides	Goethite	FeO(OH)
Sulphides	Sphalerite	ZnS
	Galena	PbS
	Pyrite	FeS_2_
	Pyrrhotite	FeS
	Digenite	(Cu,Fe)_9_S_5_
	Cubanite	CuFe_2_S_3_
	Covellite	CuS
	Chalcocite	Cu_2_S
Sulphate	Anglesite	PbSO_4_
Hydrated sulphates	Goslarite	ZnSO_4_∙7H_2_O
	Gypsum	CaSO_4_·2H_2_O
	Rapidcreekite	Ca_2_(SO_4_)(CO_3_)·4H_2_O
	Ktenasite	ZnCu_4_(SO_4_)_2_(OH)_6_·6H_2_O
	Posnjakite	Cu_4_[(OH)_6_|SO_4_]·H_2_O
Silicates	Willemite	Zn_2_SiO_4_
	Fayalite	Fe_2_SiO_4_
	Kirschsteinite	CaFe^2+^SiO_4_
	Forsterite	Mg_2_SiO_4_
Aluminosilicate	Melilites	(Ca,Na)_2_(Al,Mg)[(Si,Al)_2_O_7_]
Carbonates	Cerussite	PbCO_3_
	Smithsonite	ZnCO_3_
	Hydrozincite	Zn_5_[(OH)_3_/CO_3_]_2_
	Hydrocerussite	Pb_3_(CO_3_)_2_(OH)
Spinels	Magnetite	Fe_3_O_4_
	Hercynite	FeAl_2_O_4_
	Franklinite	ZnFe_2_O_4_
	Gahnite	ZnAl_2_O_4_
	Ulvöspinel	Fe_2_TiO_4_

**Table 3:** Parameters of the Zn-Pb slag fuming process [47].

Parameter	Unit	Value
The amount of slag processed	Mg/h	10
The amount of air for spraying and feeding the oil	Nm^3^/h	10
The amount of primary air for oil combustion	Nm^3^/h	1.53
The amount of secondary air for oxidation of vapours and afterburning of gases	Nm^3^/h	1.54
Slag temperature	K	1520–1570

**Table 4:** Chemical composition of slag and products of the fuming process [47].

Type of Material	Content (wt%)							
	Zn	Pb	Cu	S	FeO	SiO_2_	CaO	Ag (g/Mg)
Output slag	6.5	0.5	0.4	2.7	37	20	14	30
Dust	60	12	0.1	0.5	0.3	0.1	0.1	100
Waste slag	3.0	0.1	0.5	2.6	40	19	13	25

**Table 5:** Chemical composition of EAF dust (in wt%) [47].

Element	Zn	Pb	Fe	Cu	S	SiO_2_	CaO	MgO	MnO	Al_2_O_3_
Content	21.6	1.3	29.5	0.1	0.5	5.6	9.3	2.7	2.2	0.7

## Data Availability

Not applicable.
